# The antimalarial activity of the pantothenamide α-PanAm is via inhibition of pantothenate phosphorylation

**DOI:** 10.1038/s41598-017-14074-9

**Published:** 2017-10-27

**Authors:** Joy E. Chiu, Jose Thekkiniath, Jae-Yeon Choi, Benjamin A. Perrin, Lauren Lawres, Mark Plummer, Azan Z. Virji, Amanah Abraham, Justin Y. Toh, Michael Van Zandt, Ahmed S. I. Aly, Dennis R. Voelker, Choukri Ben Mamoun

**Affiliations:** 1 Section of Infectious Diseases, Department of Internal Medicine, Yale University School of Medicine, New Haven, Connecticut USA; 2 Basic Science Section, Department of Medicine, National Jewish Health, 1400 Jackson St, Denver, Colorado 80206 USA; 3 New England Discovery Partners, Branford, CT USA; 4 Department of Tropical Medicine, Tulane University School of Public Health and Tropical Medicine, New Orleans, LA 70112 USA

## Abstract

The biosynthesis of the major acyl carrier Coenzyme A from pantothenic acid (PA) is critical for survival of *Plasmodium falciparum* within human erythrocytes. Accordingly, a PA analog α-PanAm showed potent activity against blood stage parasites *in vitro*; however, its efficacy *in vivo* and its mode of action remain unknown. We developed a new synthesis route for α-PanAm and showed that the compound is highly effective against blood stages of drug-sensitive and -resistant *P. falciparum* strains, inhibits development of *P. berghei* in hepatocytes, and at doses up to 100 mg/kg also inhibits blood stage development of *P. chabaudi* in mice. We used yeast and its pantothenate kinase Cab1 as models to characterize mode of action of α-PanAm and found that α-PanAm inhibits yeast growth in a PA-dependent manner, and its potency increases dramatically in a yeast mutant with defective pantothenate kinase activity. Biochemical analyses using ^14^C-PA as a substrate demonstrated that α-PanAm is a competitive inhibitor of Cab1. Interestingly, biochemical and mass spectrometry analyses also showed that the compound is phosphorylated by Cab1. Together, these data suggest that α-PanAm exerts its antimicrobial activity by direct competition with the natural substrate PA for phosphorylation by the pantothenate kinase.

## Introduction

Malaria is an important human parasitic disease that continues to threaten the lives of ~50% of the world’s population^1^. Although control interventions implemented since year 2000 have had a major impact on malaria incidence, the disease is still responsible for more than 400,000 deaths annually^2^, with most fatalities attributed to infection by *P. falciparum* and occurring primarily in sub-Saharan Africa^2^. Chemotherapies are the main line of defense against the parasite. However, most currently approved drugs are likely to become ineffective due to the rapid and widespread escalation of resistance. This is particularly alarming in light of the growing reports of declining efficacy of Artemisinin Combination Therapies (ACTs)^3,4^. The potential failure of ACTs highlights the need for new strategies to control malaria.

The life cycle of *P. falciparum* within human red blood cells is absolutely dependent on its ability to acquire nutrients from human plasma and to utilize them for the synthesis of essential macromolecules and cofactors^5–12^. Pantothenic acid (vitamin B5), which serves as a precursor for the synthesis of Coenzyme A (CoA), has been shown to play a critical role in parasite metabolism and survival within human erythrocytes^5,8,13^. Removal of pantothenic acid from the culture medium of *P. falciparum* or treatment of the parasite with pantothenic acid analogs result in parasite death^5,8,14,15^.

**Figure 1:**
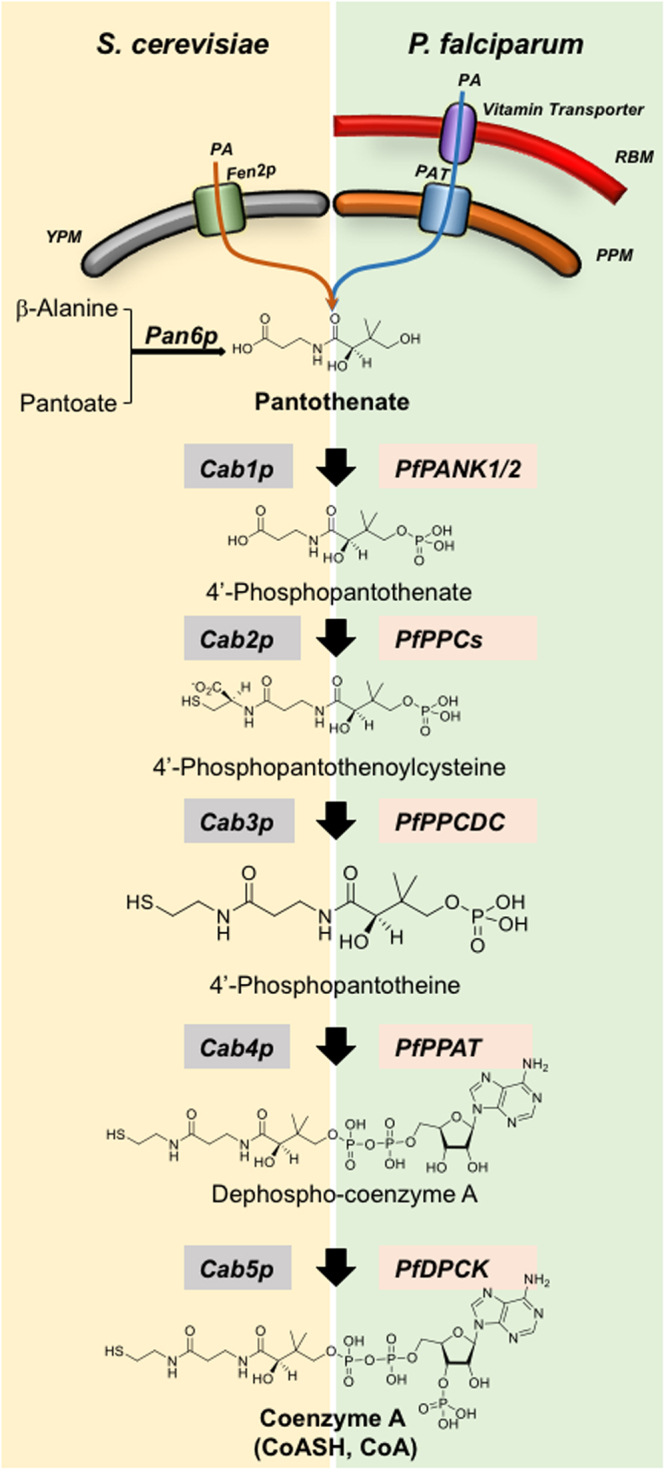
Coenzyme A biosynthesis pathways in *S. cerevisiae* and *P. falciparum*. YPM (yeast plasma membrane), RBM (red blood cell membrane), PAT (pantothenic acid transporter), PPM (parasite plasma membrane).

The synthesis of CoA from pantothenic acid (Fig. 1) begins with the transport of this precursor across the plasma membrane by a specialized pantothenate transporter. The precursor is first phosphorylated to form 4′-phosphopantothenic acid, which is then converted into 4′-phospho-N-pantothenoylcysteine and subsequently into 4′-phosphopantotheine, dephosphocoenzyme A, and CoA. In yeast, this activity is encoded by the *CAB1* gene and catalyzes the phosphorylation of pantothenate either transported from the yeast environment via the pantothenate transporter Fen2p or synthesized endogenously from β-alanine by the pantothenate synthase Pan6p^16^. Strains lacking either *FEN2* (*fen2*Δ strain) or *PAN6* (*pan6*Δ strain) are viable, whereas cells lacking the *CAB1* gene or both *FEN2* and *PAN6* are not viable.

Although pantothenate analogs have long been considered as candidates to develop new classes of antimicrobials^17–20^, identification of lead compounds with favorable pre-clinical properties has been limited by either low antimicrobial activity *in vitro*, inadequate selectivity, or lack of potency *in vivo* ^14,21^. Recent studies have demonstrated that host serum vanin enzymes reduce activity of these drugs^22^. This critical finding led to the design of pantothenamide analogs with structural modifications that render them resistant to vanin hydrolysis^23^. One such compound is alpha-methyl-N-phenethyl-pantothenamide (α-Me-N-PE-PanAm, here referred to as α-PanAm), which showed potent activity against the *P. falciparum* drug-sensitive strain 3D7^23^ and excellent selectivity (ratio of toxicity vs therapeutic effect against human HFF cell line vs *P. falciparum*, respectively).

Here we report that α-PanAm inhibits *P. falciparum* strains that are either sensitive or resistant to the antimalarials pyrimethamine and chloroquine. Furthermore, using murine malaria models, we show that α-PanAm inhibits blood and liver stage development of *Plasmodium* parasites. Interestingly, we also found that α-PanAm inhibits growth of the yeast *Saccharomyces cerevisiae*, and this antimicrobial activity is dramatically enhanced in a mutant with a temperature sensitive pantothenate kinase. Biochemical analyses demonstrated that α-PanAm inhibits pantothenate phosphorylation.

## Results

### Stage specificity and *in vivo* efficacy of α-PanAm

**Figure 2:**
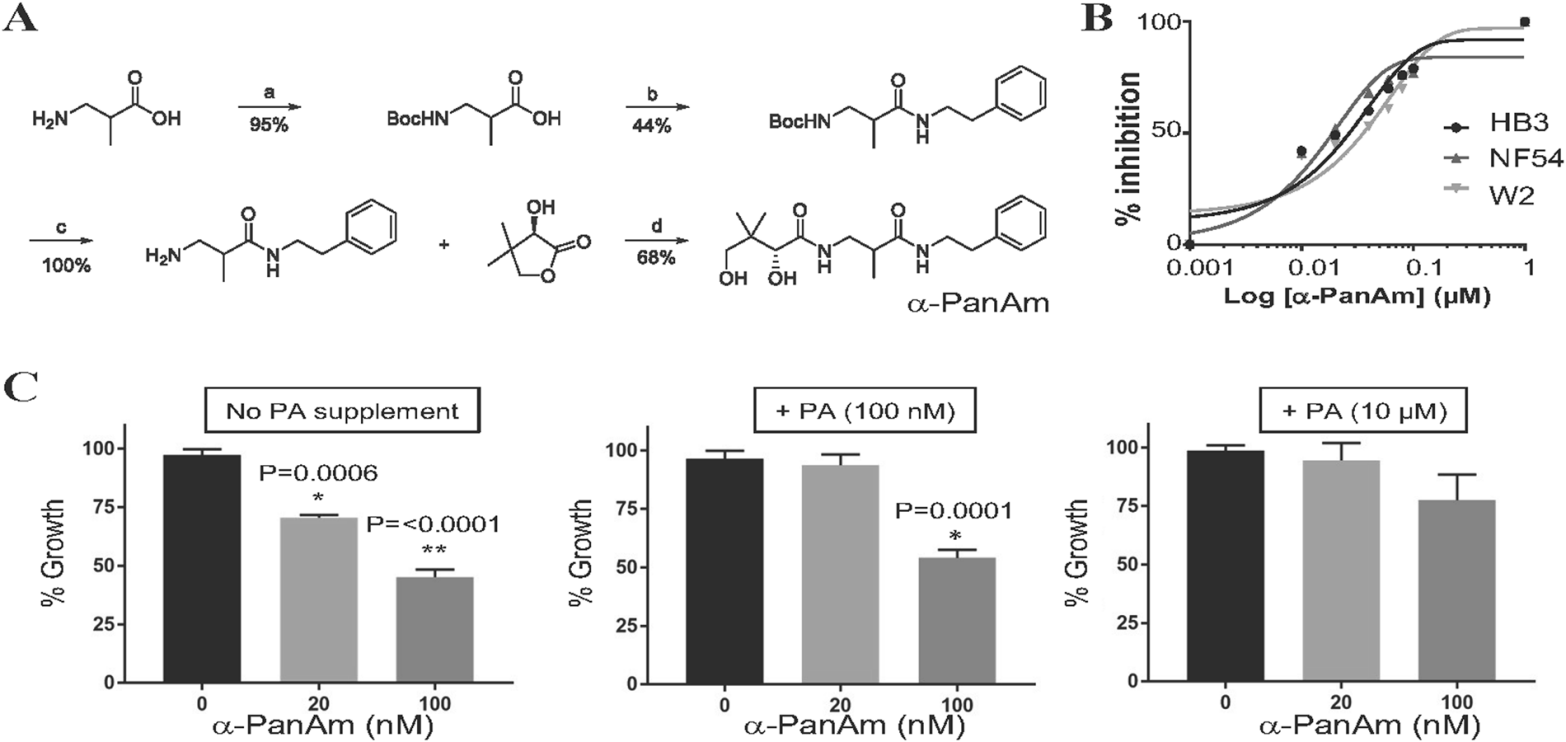
α-PanAm inhibits growth of drug-sensitive and drug-resistant *P. falciparum* strains in a pantothenic acid-dependent manner. (**A**) Chemical synthesis route for α-PanAm. The 4 steps (a, b, c and d) of synthesis are described in details in Material and Methods. (a) di-*tert*-butyl dicarbonate, 1,4-dioxane, aq. NaOH, 5–25 °C. (b) phenethylamine, EDCI, pyridine, THF, 0 °C. (c) TFA, DCM, 25 °C. (d) methanol, 40 °C, 16 h. (**B**) growth curve of *P. falciparum* NF54, HB3 and W2 in human erythrocytes on complete RPMI medium lacking or supplemented with increasing concentrations of α-PanAm. (**C**) Growth of *P. falciparum* (NF54 strain) in human erythrocytes in complete RPMI medium (1.14 µM pantothenic acid) containing albumax supplemented with increasing concentrations of pantothenic acid (PA) and α-PanAm. Starting parasitemia was 1%. (**D**) Stage specificity of inhibition of *P. falciparum* by α−PanAm, *P. falciparum* culture was initiated at 1% starting parasitemia in human erythrocytes and α-PanAm applied at 0, 12 and 24 h. Parasite stages were monitored by light microscopy after Giemsa staining.

The synthesis of α-PanAm (Fig. 2A) was achieved using 1-ethyl-3-(3-dimethylaminopropyl)carbodiimide (EDCI) coupling of N-tert-Butyl carbamate (*N*-Boc) protected 3-aminoisobutyrate with phenethylamine and subsequent reaction with pantolactone. Unlike the previously reported method^23^, which requires cation-exchange chromatography, this modified process utilized only normal phase chromatography making it better suited for new analog synthesis and future large scale production. The structure and purity of α-PanAm was confirmed by NMR and mass spectrometry. To examine the efficacy of the compound against *P. falciparum*, increasing concentrations of the compound were added to cultures of the drug-sensitive NF54 (chloroquine IC_50_ ~ 11 nM, pyrimethamine IC_50_ ~ 18 nM), pyrimethamine-resistant HB3 (chloroquine IC_50_ ~ 8 nM, pyrimethamine IC_50_ ~ 500 nM), and chloroquine-resistant W2 (chloroquine IC_50_ ~ 90 nM, pyrimethamine IC_50_ ~ 1.5 µM) strains^24,25^. The strains were inhibited by the compound with IC_50_ ranging between 20 nM and 30 nM (Fig. 2B and Table 1). Since α-PanAm is an analog of PA, we also examined its activity in the presence of increasing concentrations of exogenous PA. As shown in Fig. 2C, increasing concentrations of vitamin B5 reduced the efficacy of α-PanAm.

**Table 1:** IC_50_ concentrations (nM) of α-PanAm, chloroquine and pyrimethamine in *P. falciparum* strains NF54, HB3 and W2.

	NF54	HB3	W2	Reference
α-PanAm	20 ± 0.47	20 ± 3.12	30 ± 0.65	This work
Chloroquine	11 ± 2.1	8 ± 1.4	90 ± 3.7	24,25
Pyrimethamine	18 ± 0.8	500 ± 45	1500 ± 5.8	24

**Figure 3:**
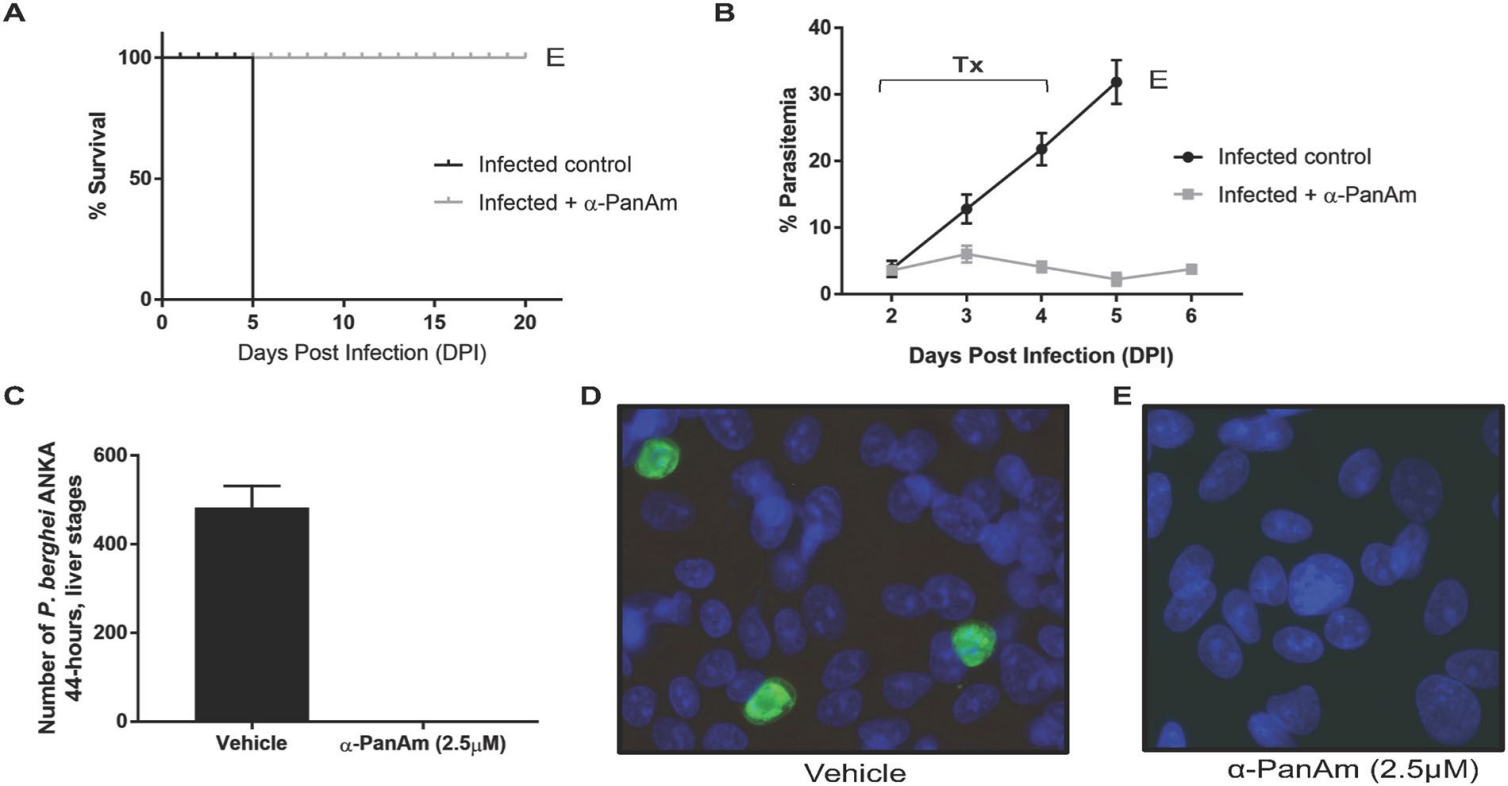
α-PanAm treatment inhibits the growth of rodent malaria blood and liver stage parasites. (**A**) Survival curves for mice either sham treated (saline) or α-PanAm-treated (40 mg/kg). All untreated mice were moribund at day 5 after infection and were immediately euthanized. All treated animals live until experimental endpoint of day 20. **(B)** Daily average blood stage parasitemia (% infected erythrocytes out of >5000 cells counted) in two groups of Swiss Webster mice infected IV by injection of 10^6^ infected erythrocytes of *P. chabaudi* AS strain. Treatment of one group with α-PanAm (daily IP injection of 40 mg/kg dose per mouse) and with 10% DMSO/PBS for the other group on days 2, 3 and 4 after parasite infection confirms inhibition of parasite blood stage growth caused by the drug treatment (Tx). All mice of the control group started to show signs of pathology on day 4 after infection and were euthanized (**E**) on DPI5. At the time of the treatment and tail blood smearing, parasites detected were mostly early trophozoites/late rings. (**C**) Average number of *P. berghei* ANKA strain liver stage parasites developing in HepG2 Hepatoma cells 44 hrs after the invasion of 25,000 salivary gland sporozoites per well. Treatment with 2.5 µM.α-PanAm started at 2 hrs after sporozoite invasion, and the control wells received medium with 1% DMSO/DMEM. (**D**,**E**) Representative images of parasitized HepG2 cells treated with either vehicle (**D**) or α-PanAm (**E**).

To assess the *in vivo* activity of the compound, two groups of three mice each were infected with 10^6^ blood stage parasites of *P. chabaudi chabaudi* AJ strain by intravenous injection. When average parasitemia of both mouse groups reached about 3% at day 2 post infection (DPI2), the mice were intraperitoneally injected with either saline (control group) or 40 mg/kg α-PanAm per day for three consecutive days. Control mice infected with the parasite showed signs of pathology at DPI4 and were euthanized at DPI5 with average blood parasitemia over 30%. In contrast, no signs of pathology were seen in α-PanAm-treated mice infected with *P. chabaudi* and average blood parasitemia remained at ~2% **(**Fig. 3A,B **)**. With no further treatment, mice were subsequently euthanized at DPI20 due to high parasite load. Treatment with α-PanAm at 100 mg/kg resulted in a 15-fold lower parasitemia, but did not achieve radical cure.

To assess α-PanAm antimalarial activity at other malaria parasite life cycle stages, we examined the ability of the compound to inhibit parasite development within hepatocytes. In 8-well chamber slides, 2.5 × 10^4^ salivary gland sporozoites of *P. berghei* ANKA strain (day 18 post mosquito feeding) were added to 40,000 HepG2 hepatoma cells (seeded 2 days prior to the assay) per well. At 2 hours after sporozoite invasion, 2.5 µM α-PanAm was added to half of the wells, while the control wells received complete cell culture medium with the respective amount of saline with 1% DMSO. Cells were collected 44 hours following sporozoite invasion, fixed, permeabilized and incubated with anti-HSP70 and secondary green fluorescent antibodies. The infected wells had an average of 483 fully developed liver stage parasites (Fig. 3C,D). In contrast, the α-PanAm treated wells did not have any mature fully developed liver stages of *P. berghei* ANKA strain (Fig. 3C,E). Together, these data demonstrate that α-PanAm exhibits antimalarial activity against *in vivo* blood stages and *in vitro* liver stages of rodent malaria parasites.

### Pantothenate-dependent inhibition of yeast growth by α-PanAm

**Figure 4:**
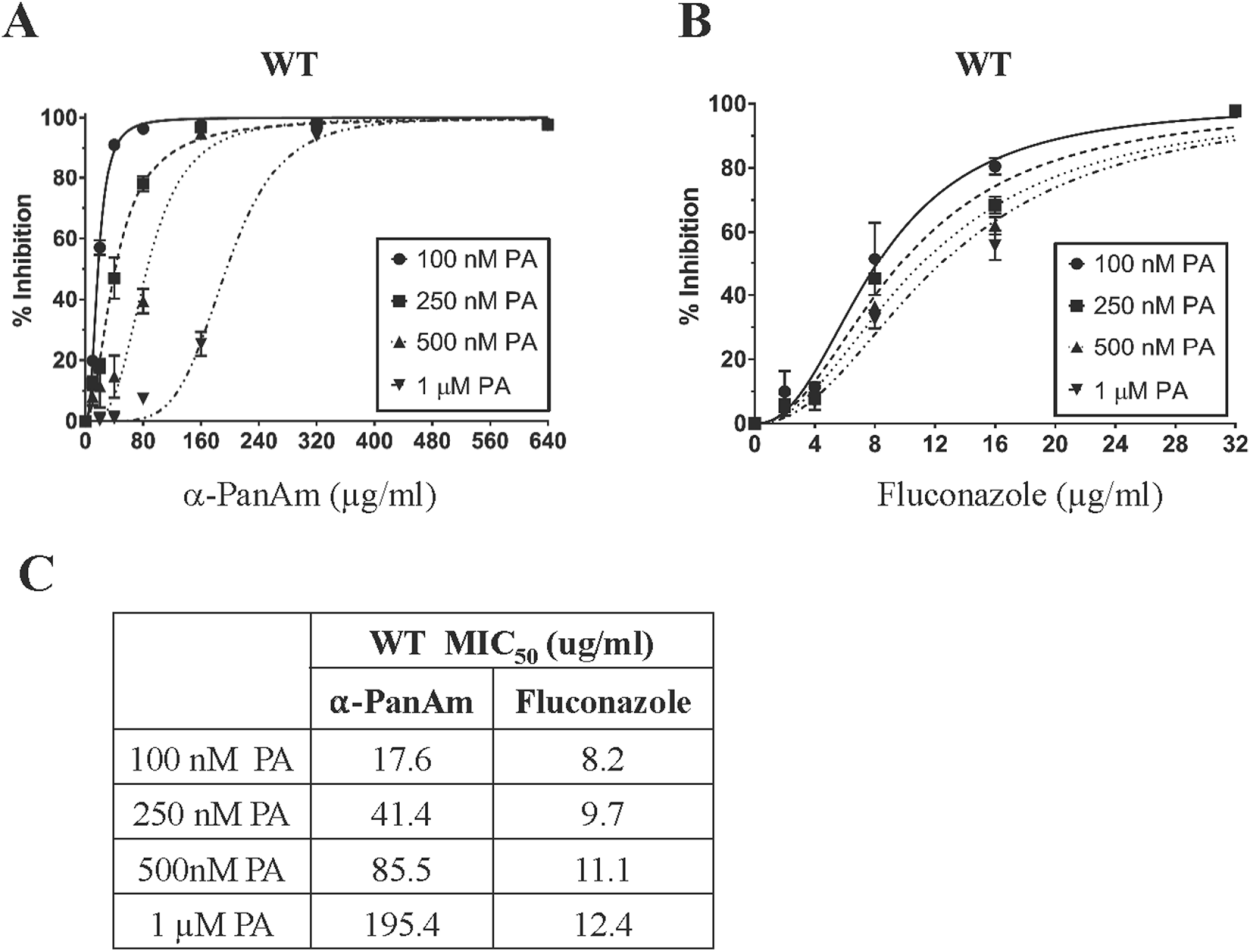
Sensitivity of wild type *S. cerevisiae* to α-PanAm. (**A**) Growth inhibition of wild type yeast cells in pantothenic acid-free medium supplemented with 100 nM, 250 nM, 500nM and 1 µM, pantothenic acid (PA) in the absence or presence of increasing concentrations of α**-**PanAm. (**B**) Growth inhibition of wild type cells on pantothenic acid-free medium supplemented with 100 nM, 250 nM, 500 nM, or 1 µM pantothenic acid (PA) in the absence or presence of increasing concentrations of fluconazole. (**C**) MIC_50_ of α-PanAm and fluconazole on wild type yeast cells in different concentrations of pantothenic acid.

Pantothenate kinases catalyze the first step in the synthesis of CoA by phosphorylating PA into phospho-pantothenate (P-PA)^26^. *P. falciparum* encodes two homologs of eukaryotic pantothenate kinases *PfPanK1* and *PfPanK2* ^27^. Although these two genes have been recognized as candidate pantothenate kinases since the completion of the *P. falciparum* genome sequence, all attempts to demonstrate activity of the encoded enzymes *in vitro* have not been successful. Similarly, expression of codon-optimized PfPanK1, PfPanK2 or both in the yeast mutant *cab1* ^*ts*^ that carries a thermosensitive allele of the pantothenate kinase gene *CAB1*, did not complement the growth defect of the mutant at 37 °C. Therefore, we used yeast as a model system to assess possible antifungal activity of α-PanAm and elucidate its mode of action. The yeast Cab1 enzyme shares 27% identity and 50% similarity with PfPanK1, and 29% identity and 43% similarity with PfPanK2. Wild type yeast cells grown in the absence or presence of increasing concentrations of α-PanAm up to 640 µg/ml showed a dose dependent inhibition of growth. Interestingly, the MIC_50_ of the compound was found to be significantly affected by the availability of PA in the medium. Whereas at 100 nM PA, the MIC_50_ was ~18 µg/ml, the MIC_50_ of α-PanAm increased to ~41 µg/ml at 250 nM PA, 86 µg/ml at 500 nM PA and 195 µg/ml in the presence of 1 µM PA (Fig. 4A and C). This decrease in sensitivity to the compound with increasing PA availability suggests that the mode of action of the compound in yeast and *P. falciparum* might be similar. As a control, the MIC_50_ of the well-known antifungal drug fluconazole was only marginally affected by exogenous pantothenic acid with the MIC_50_ value of fluconazole changing by only 1.5-fold from 8.2 µg/ml at 100 nM PA to 12.4 µg/ml in 1 µM PA compared to 10-fold seen for α-PanAm (Fig. 4B,C).

**Figure 5:**
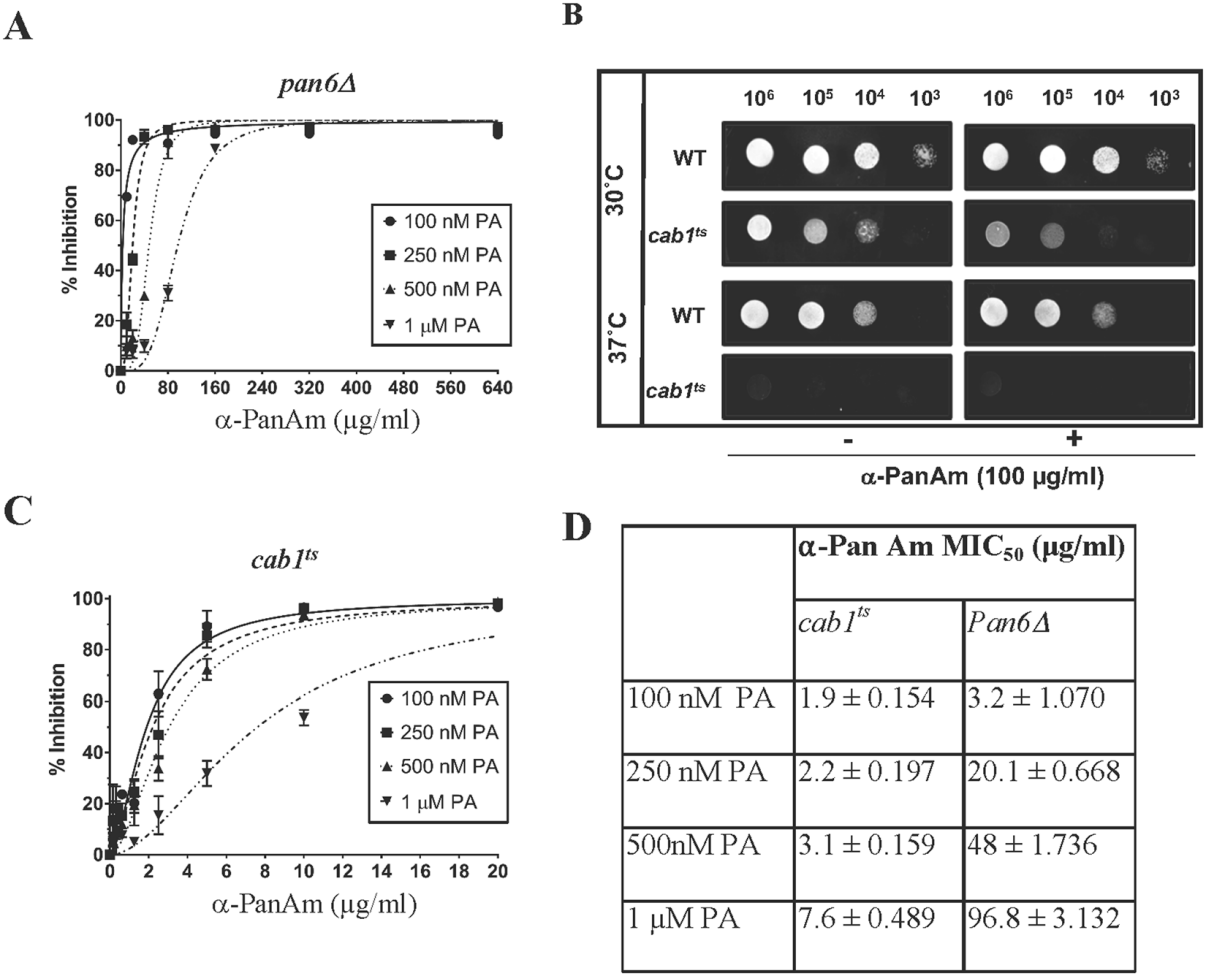
Sensitivity of yeast *pan6*Δ and *cab1* ^*ts*^ strains to α-PanAm. *pan6*Δ (**A**) and *cab1* ^*ts*^ (**C**) yeast cells were inoculated at 10^4^ cells/ml in pantothenic acid-free medium lacking or supplemented with 100 nM, 250 nM, 500 nM, and 1 µM pantothenic acid (PA) in the absence or presence of increasing concentrations of α-PanAm. (**B**) Growth of WT and *cab1* ^*ts*^ strains on YPD agar plates lacking or supplemented with 100 µg/ml α-PanAm at 30 °C and 37 °C. Limiting dilution was performed by spotting serial 10-fold dilutions of *WT* or *cab1* ^*ts*^ cells. Plates were incubated at 30 °C and 37 °C and imaged at 26 hrs after inoculation. (**D**) MIC_50_ of α-PanAm on *pan6*Δ and *cab1* ^*ts*^ yeast cells in different concentrations of pantothenic acid.

Yeast cells, unlike *P. falciparum*, can synthesize pantothenate *de novo* from β-alanine using the pantothenate synthase Pan6p (Fig. 1). We therefore examined the sensitivity of *pan6*Δ cells lacking the *PAN6* gene to α-PanAm (Fig. 5A). Consistent with the essential role of this pathway, the *pan6*Δ mutant did not grow in the absence of pantothenic acid. Interestingly, compared to wild type, *pan6*Δ mutants were 6x more sensitive to α-PanAm at 100 nM PA and twice as sensitive as WT at higher PA concentrations (Fig. 5A,D).

### Yeast cells altered in Cab1p activity show increased sensitivity to α-PanAm

The PA-dependent sensitivity of WT cells to α-PanAm and the increased sensitivity of yeast cells to inhibition in the absence of the endogenous pathway support the model that the compound targets pantothenate phosphorylation encoded by the *CAB1* gene^16^. To genetically evaluate this hypothesis, we examined the sensitivity of a yeast mutant *cab1* ^*t*s^ carrying a thermosensitive mutation in the *CAB1* gene^16^. The growth of the mutant is blocked at 37 °C and is significantly reduced with low PA concentrations at 30 °C (Fig. 5B). We found that the *cab1* ^*ts*^ mutant was highly sensitive to α-PanAm with an MIC_50_ value of 1.9 µg/ml at 100 nM PA and 7.6 µg/ml at 1 µM PA, representing 9 × and 26 × higher sensitivity of the strain to the compound compared to WT, respectively (Fig. 5C,D).

### α-PanAm inhibits Cab1p pantothenate kinase activity

**Figure 6:**
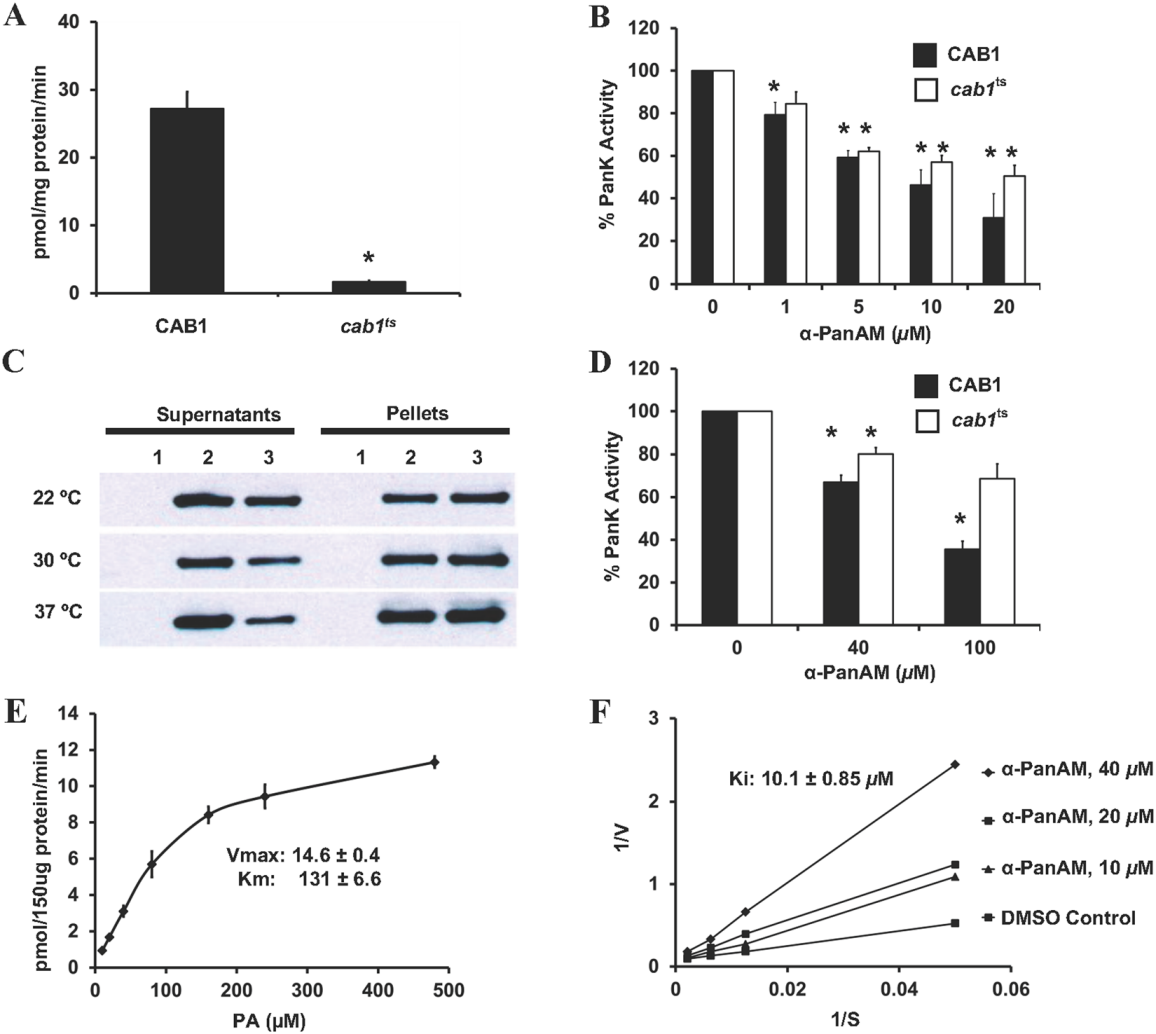
α-PanAm inhibits Cab1p pantothenate kinase activity (radioactive assay). (**A**) PanK activities were measured in 72 µg of yeast cell free extract proteins from WT and *cab1* ^*ts*^ strains using 50 µM D-[1-^14^C] pantothenate as a substrate at the permissive temperature of 30 °C, for 10 min. (**B**) PanK activities were measured in the presence of indicated concentrations of α-PanAm and the relative enzyme activities are presented as the percentages relative to the activity of DMSO control (100%). (**C**) Epitope-tagged His_6_-CAB1 and His_6_-cab1^G351S^ fusion proteins were expressed in *E. coli*. The proteins were induced overnight at 22 °C, 2.5 hrs at 30 °C, or 2 hrs at 37 °C. Solubility of the His_6_-CAB1and His_6_-cab1^G351S^ was analyzed by western blot using an anti- His_6_ antibody. (**D**) The PanK activities of purified His_6_-CAB1 and His_6_-cab1^G351S^ mutant proteins were measured in the presence of the indicated concentrations of α-PanAm. The relative enzyme activities are shown. (**E**) V_max_ and K_m_ values of the yeast PanK enzyme were determined after conducting the enzyme assay with 150 µg of yeast cell free extracts from the WT strains with varying pantothenate concentrations. Reactions were performed at 30 °C for 10 minutes. (**F**) Inhibition of Cab1p enzyme activity by α-PanAm was investigated by conducting enzyme assay as a function of varying pantothenate and α-PanAm concentrations. Double reciprocal plots of the data obtained were used to determine K_i_ values. V_max_, *K*_m_, and K_i_ values were determined using the Prism software. Data are expressed as mean ± SE of three experiments. The asterisk indicates P < 0.05 as compared to control.

To assess direct inhibition of pantothenate phosphorylation (PanK) activity by α-PanAm, cell extracts from wild type and *cab1* ^*ts*^ were used to measure native enzyme activity with a^14^C-PA substrate in the absence or presence of increasing concentrations of the compound. As shown in Fig. 6A, cell extracts prepared from the *cab1* ^*ts*^ mutant had only ~6% of wild type PanK activity even at the permissive temperature of 30 °C. Previous studies have indicated that the phenotype of the *cab1* ^*ts*^ mutant was due to a mutation G351S in the Cab1 enzyme^16^. Therefore, we cloned the wild type and mutated *CAB1* gene into an *E. coli* expression vector, purified the recombinant proteins, and determined their activity *in vitro* at different temperatures, in the absence or presence of α-PanAm. Separation of cell free extracts at 20,000 × g showed equal amounts of wild type Cab1p associated with the soluble fraction at 22 °C, 30 °C and 37 °C, whereas the Cab1^G351S^ recombinant protein present in the soluble fraction decreased with increasing temperature (Fig. 6C). PanK activity measured using yeast cell extracts or purified soluble enzyme (Fig. 6B,D) showed sensitivity of the enzymes to α-PanAm even though the purified epitope-tagged enzyme was less sensitive to the inhibitor. This finding suggests that the increased sensitivity of *cab1* ^*ts*^ mutant at 30 °C is not due to increased sensitivity of the mutated enzyme but rather due to an altered folding state of the enzyme rendering only a small fraction of the enzyme capable of phosphorylating pantothenate. To further understand the kinetics of Cab1p, enzyme activity was measured in presence of varying concentrations of PA, and *V*_*max*_ and *K*_m_ values were determined (Fig. 6E). The apparent *K*_m_ of Cab1p in the presence of excess ATP (2.5 mM) was 131 ± 6.6 µM. Previous studies of prokaryotic and eukaryotic pantothenate kinase enzymes have reported apparent *K*_*m*_ values for pantothenate between 5.5 µM and 621 µM^28^. Kinetic analyses of α-PanAm inhibition of Cab1p are shown in Fig. 6F, using different concentrations of pantothenate, and three different concentrations of α-PanAm. α-PanAm did not alter the *V*_*max*_ but increased the *K*_*m*_ for PA, demonstrating competitive inhibition of the Cab1p enzyme. The *K*_*i*_ of α-PanAm was determined to be 10.07 ± 0.85 μM. The inhibitor exhibits about 13-fold higher affinity toward the Cab1p enzyme compared to the natural substrate, PA.

**Figure 7:**
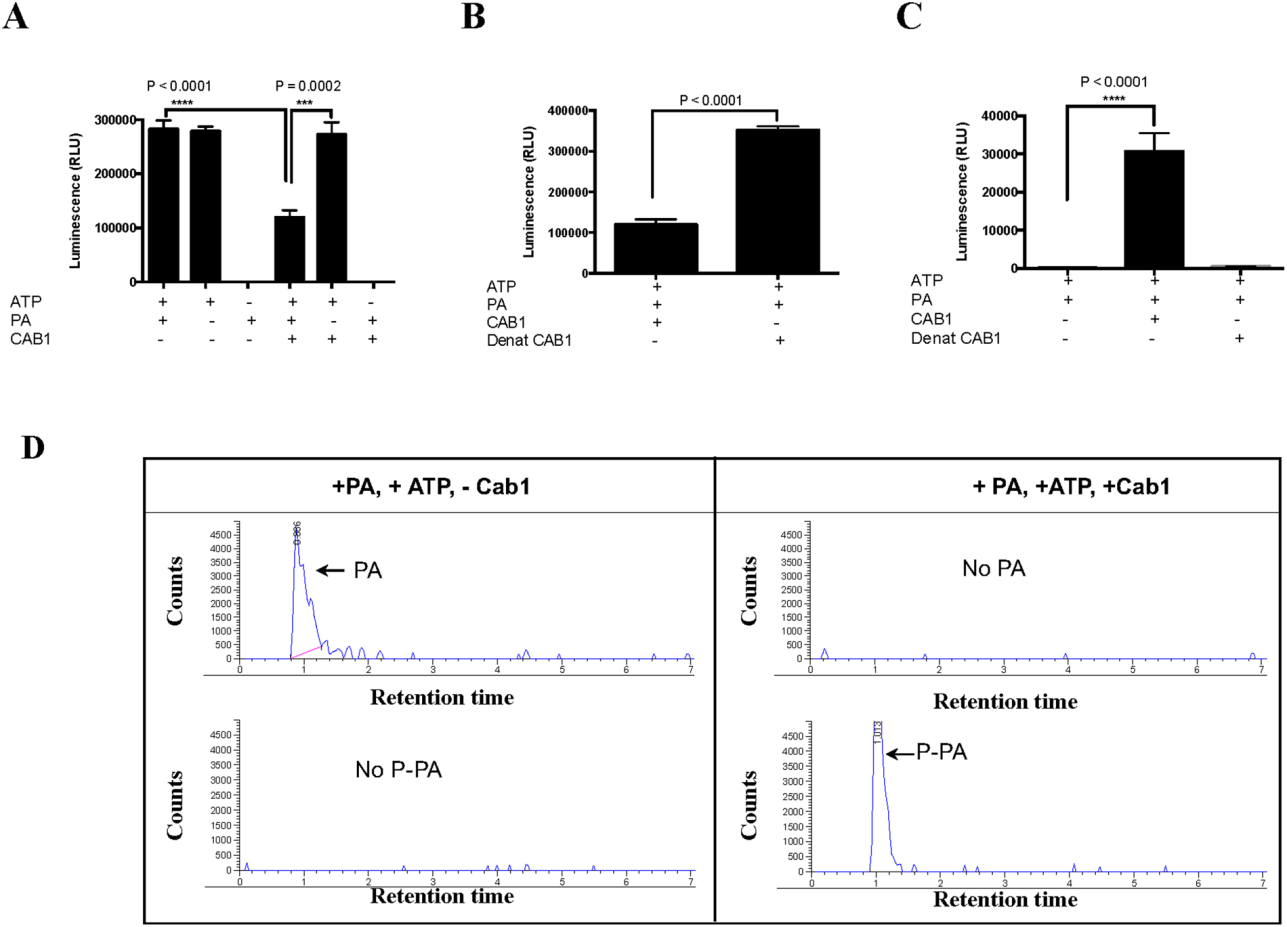
Analysis of Cab1 pantothenate kinase activity. (**A**–**C**) Cab1 enzyme activity as a function of ATP consumption (**A** and **B**) or ADP production (**C**). The amount of ATP in the reaction was measured using the Kinase-Glo Plus Luminescent Kinase (Kinase-Glo) assay. In this reaction, the luminescence signal (Relative light units- RLU) is inversely correlated with kinase activity. Kinase-Glo Reagent was added following a kinase reaction and incubated for 60 min at room temperature. Reactions were carried out in the presence or absence of ATP, PA and Cab1, and luminescence determined using a BioTek SynergyMx Microplate reader (**A**). Heat inactivated Cab1 (Denat Cab1) is used as a control (**B**). Cab1 activity as a function of ADP production was measured using the ADP-Glo Kinase assay. In this reaction, luminescence (RLU) is directly correlated with the kinase activity. Kinase reactions were carried out in the presence of active or heat inactivated Cab1 (**C**). Data are expressed as the mean ± SE of four experiments. (**D**) Mass spectrometry analysis of Cab1 pantothenate phosphoprylation activity. Reactions were conducted in the presence of PA and ATP and in the presence (+Cab1) or absence (-Cab1) of Cab1. Upper panels show negative ion scans specifically looking for the mass of PA-1 (mass range 217–219). The lower panels show negative ion scans specifically looking for the mass of P-PA-1 (mass range 297–299). The y-axis was normalized to 5000 units for comparison purposes.

We further evaluated the activity of Cab1 using Kinase-Glo and ADP-Glo assays which measure luciferase activity as a direct reporter of the amount of ATP consumed and ADP produced, respectively. As shown in Fig. 7, addition of Cab1 reduces ATP content (Fig. 7A) and increases ADP amounts (Fig. 7C) in the presence of PA. As a control, heat inactivation of the enzyme results in high luciferase activity (Fig. 7B). Mass spectrometry analysis of the reaction confirmed the substantial presence of the mass of phosphopantothenate being formed in the presence of the enzyme while only PA is present in the Cab-1 control. (Fig. 7D).

**Figure 8:**
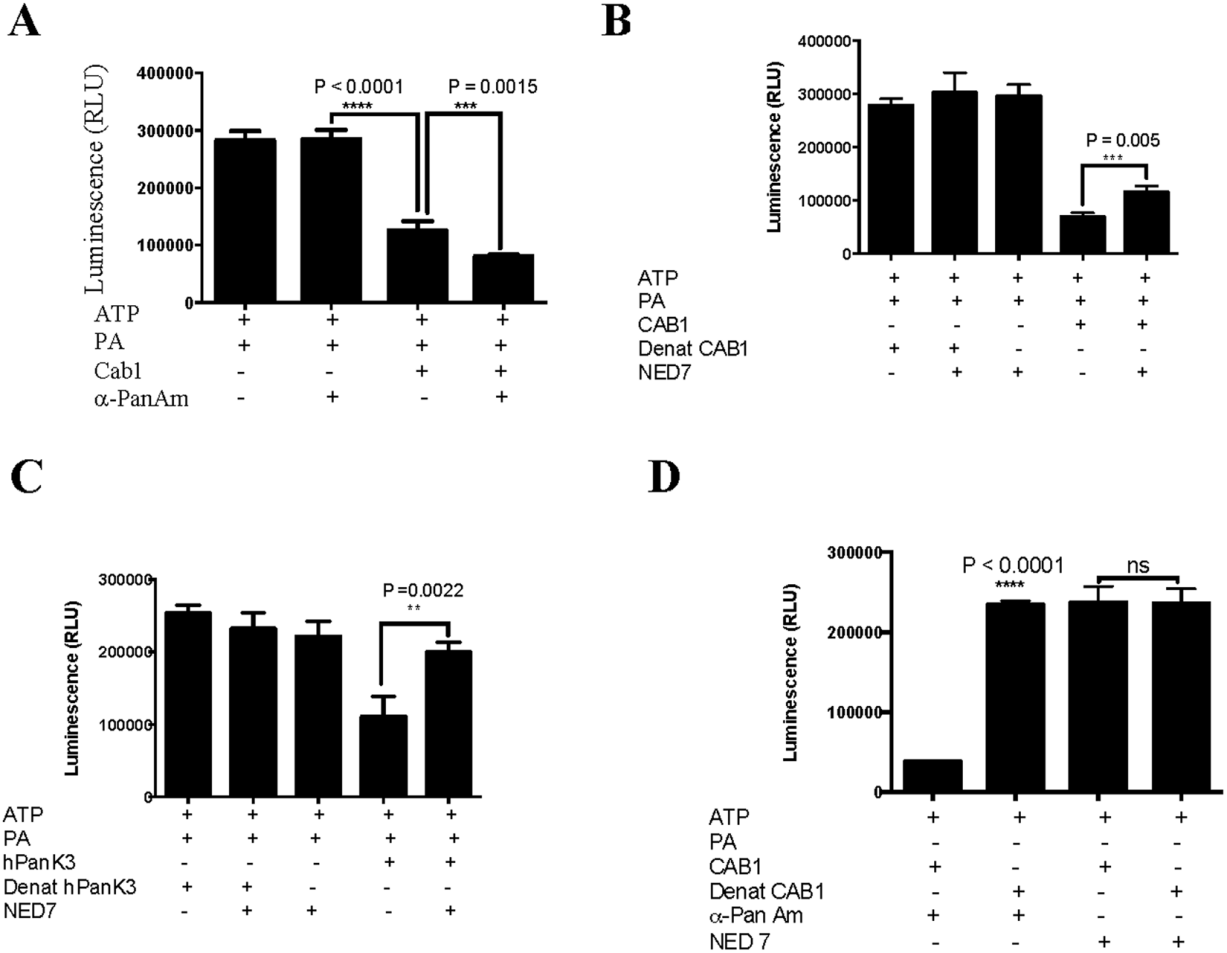
Phosphorylation of α-PanAm by Cab1. (**A**) Cab1 activity was measured in the absence or presence of α-PanAm (10 µM) using the Kinase-Glo assay, which measured the amount of ATP at the end of the reaction. (**B** and **C**) Inhibition of Cab1 (**B**) and human PanK3 (**C**) activity by 3-(2,4-dimethylpyrido- [2′,3′:3,4]pyrazolo[1,5-a]pyrimidin-3-yl)-N-(3-(methylthio)phenyl) propanamide (NED7: 10 µM). Kinase reactions were carried out in the presence of active or heat inactivated enzymes. (**D**) Phosphorylation of α-PanAm by active Cab1 enzyme. Enzymatic reactions were performed in the absence of PA but in the presence of α-PanAm or NED7. Heat inactivated Cab1 was used as a control. Data are expressed as the mean ± SE of four experiments.

**Figure 9:**
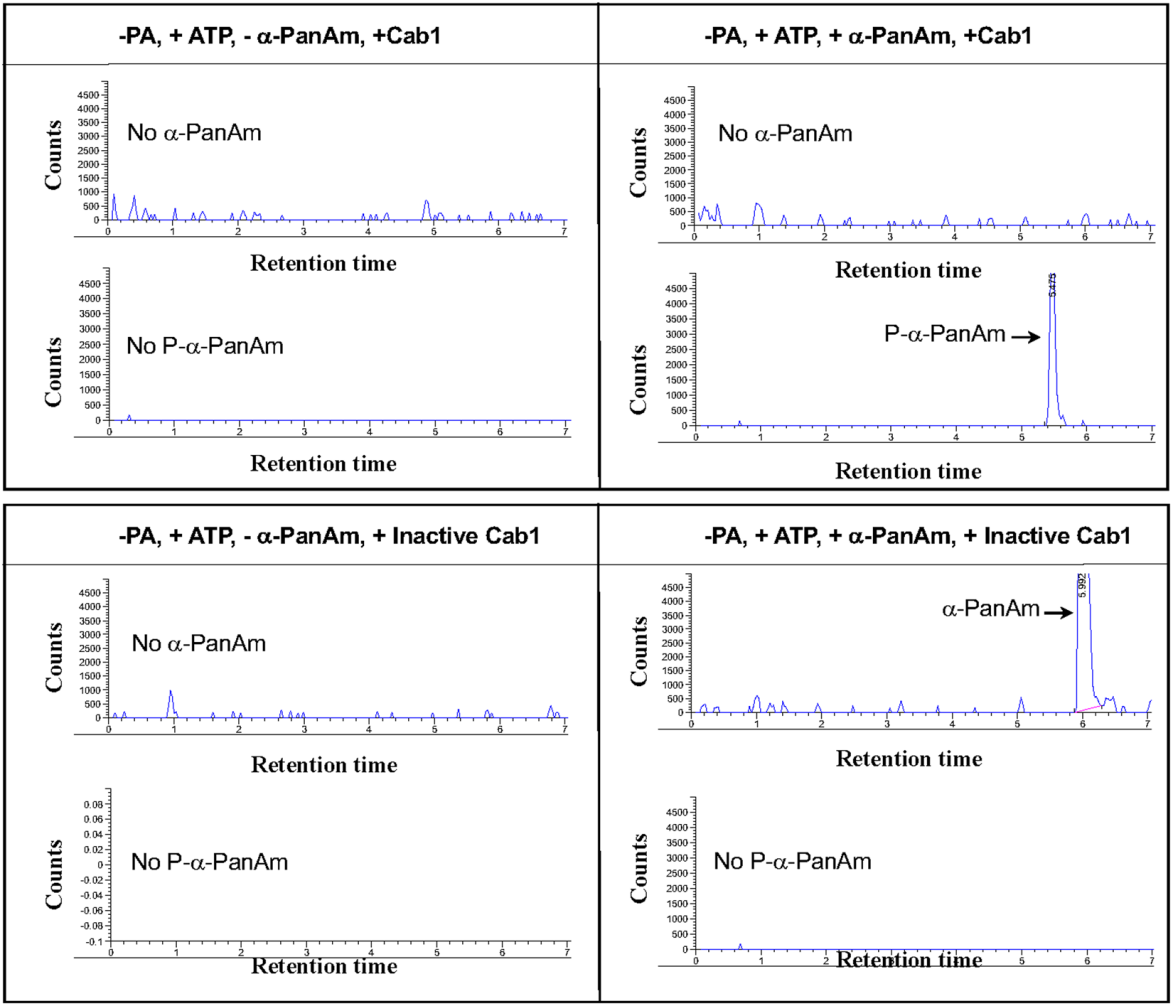
Validation of α-PanAm phosphorylation by Cab1 using Mass Spectrophotometry analysis. Mass spectrometry analysis of the kinase reaction showing only the presence of P-α-PanAm with +ATP and +Cab-1 enzyme. α-PanAm phosphorylation occurs with Cab1 (upper pair of panels) but not with denatured Cab1 (lower pair of panels) using mass spectrophotometry analysis. Upper individual panels are positive ion scans specifically looking for the mass of α-PanAm + 1 (mass range 336–338). The lower panels are negative ion scans specifically looking for the mass of P-PA-1 (mass range 297–299). The y-axis was normalized to 5000 units for comparison purposes.

Interestingly, addition of α-PanAm consistently resulted in increased ATP depletion in the assay in the presence of active Cab1 (Fig. 8A). As a control, the inhibitor 3-(2,4-dimethylpyrido-[2′,3′:3,4]pyrazolo[1,5-a]pyrimidin-3-yl)-N-(3-(methylthio)phenyl) propanamide (NED7) previously shown to inhibit human PanK3^29^ resulted in increased luciferase activity in the presence of Cab1 (Fig. 8B,C). This finding suggests that α-PanAm might be phosphorylated by Cab1 resulting in increased consumption of ATP. We therefore measured the effect of the compound on luciferase activity in the absence of pantothenic acid but in the presence of active or heat inactivated Cab1 or human Pank3. As shown in Fig. 8D, major ATP depletion occurred when α-PanAm was mixed with active Cab1, but not when mixed with heat-inactivated Cab1. Mass spectrometry analysis detected the mass of phosphorylated α-PanAm (P-α-PanAm) in the presence of active Cab1. When the Cab1enzyme was denatured only the mass of α-PanAm was detected (Fig. 9).

## Discussion

In this study, we describe a new method for synthesis of α-PanAm, and provide evidence that it is a potent inhibitor of intraerythrocytic *P. falciparum* development. We show that the compound inhibits growth of both drug-sensitive and chloroquine- and pyrimethamine-resistant parasites with IC_50_ values ranging between 20 and 30 nM. Interestingly, the potency of the compound diminished with increasing concentrations of exogenous PA, suggesting that the antimalarial activity is directly linked to the metabolism of PA. It was previously shown that this compound is selective with a selectivity index of >1500^23^. The ability of the compound to inhibit parasite development *in vivo* was assessed using the *P. chabaudi* murine model of malaria. This parasite has a nearly synchronized blood stage cycle of 24 hours and preferentially infects normocytes^30^. This parasite causes fulminant infection that results in death within 5 days. We found that mice treated for 3-days with α-PanAm at 40 mg/kg or 100 mg/kg had parasitemia levels at day 5 at ~2%, 15 times lower than vehicle treated control mice. Although these mice lived 15 days longer than control mice, the data clearly show that α-PanAm treatment does not result in cure as parasite burden increases following drug removal, resulting in death. Future studies are needed to determine whether the lack of radical cure by α-PanAm in mice is a result of a cytostatic effect of the compound *in vivo* or is due to poor pharmacological properties. Noteworthy, previous studies have shown that mice have higher PA concentrations in blood compared to humans.

Another important finding of this study is that in addition to its inhibition of blood stage development, α-PanAm treatment results in complete inhibition of *P. berghei* development in hepatocytes. This suggests that this class of compounds has the potential to achieve multistage efficacy, a favorable property for new antimalarials.

In addition to the antimalarial activity of α-PanAm, the compound was also found to inhibit yeast growth. As in *P. falciparum*, the activity of the compound against yeast cells was significantly affected by exogenous PA. The MIC_50_ of the compound in WT yeast ranged from 17.6 µg/ml at 100 nM PA, to 41.4 µg/ml at 250 nM PA, to 85.5 µg/ml at 500 nM PA, and to 195.4 µg/ml at 1 µM PA. Interestingly, under these same conditions the compound was even more effective against the *pan6*Δ and *cab1* ^*ts*^ mutants, which either lack the endogenous source of pantothenic acid or express a weak pantothenate kinase activity. These findings suggest a competitive mode of inhibition of pantothenate binding to the kinase by α-PanAm. Consistent with this hypothesis, our biochemical analyses using ^14^C-PA and cell extracts from yeast and *E. coli* expressing Cab1p and Cab1^G351S^, as well as purified Cab1p and Cab1^G351S^, demonstrated direct inhibition of pantothenate phosphorylation by α-PanAm, with a *K*_*i*_ of 10 μM. We further employed a luciferase-based assay to monitor Cab1 activity by quantifying ATP. Using this assay, we found that in the presence of PA and α-PanAm, the amount of ATP in the Cab1 reaction was 36% lower than in the presence of PA alone. Interestingly, we found that the amount of ATP in the reaction also decreased in the presence of α-PanAm even when PA was not included in the reaction. Mass spectrometry analysis confirmed a substantial mass signal corresponding to phosphorylated α-PanAm was produced during this reaction, strongly suggesting that like PA, the compound is also phosphorylated by Cab1 and thus competes directly with the natural substrate for phosphorylation. These data are consistent with a recent report by de Villiers and colleagues^31^.

The *P. falciparum* PfPanK1 and PfPanK2 share several conserved motifs with the yeast Cab1. For example, the putative ATP binding site DIGGTLAKVVFS in Cab1 is similar to the DIGGTLIKVVYV found in PfPanK1. In addition, the DMLVGDIYGT and GLKSSAIASSFG motifs found in Cab1p are similar to the DMTVQDIYGT and GLCKDLTASFFG found in PfPanK2. Whereas a direct inhibition of *P. falciparum* PfPanK1 or PfPanK2 activity has not yet been demonstrated *in vitro*, the findings using the yeast enzyme Cab1p predict that the same mechanism of inhibition by α−PanAm occurs in the parasite. We recognize that while inhibition of the endogenous parasite activity from total *P. falciparum* extracts or after immunoprecipitation can be determined, these studies are not conclusive, for they cannot specifically indicate whether the endogenous activity is due to one of the two enzymes or to a larger protein complex that includes the two enzymes.

By providing definitive evidence for α-PanAm’s inhibition of *Plasmodium* development, yeast growth, and pantothenate phosphorylation, our findings now provide a rational basis for optimizing pantothenamides using Cab1 as a surrogate enzyme. Recent genetic studies using the murine malaria parasite *Plasmodium yoelii* demonstrated an essential role of the putative malaria PanKs in parasite transmission to the mosquito^23^. This suggests that pantothenamide analogs may satisfy the desirable multistage efficacy criteria of new antimalarials to target not only blood stage development but also transmission to the mosquito.

## Material and Methods

### Synthesis of α-PanAm: Step 1: synthesis of 3-((tert-butoxycarbonyl)amino)-2-methylpropanoic acid

A suspension of beta-amino isobutyric acid (5 g, 48.5 mmol) and di-*tert*-butyl dicarbonate (10.5 g, 48.3 mmol) in 1,4-dioxane (90 mL) and water (45 mL) was cooled to 5 °C in an ice bath and carefully treated with 2 N sodium hydroxide (60.6 mL, 121 mmol) in three portions over 30 min. After the addition was completed, the solution was stirred for an additional 1 h, acidified with 2 N HCl to pH 3 and concentrated under reduced pressure to approximately 1/3 of its original volume. Following addition of saturated sodium chloride, the mixture was extracted with ethyl acetate (3x). The combined organic layers were washed with saturated sodium chloride, dried over magnesium sulfate, filtered and concentrated to give 3-((tert-butoxycarbonyl)amino)-2-methylpropanoic acid (9.35 g, 95% yield) as a colorless oil that solidified upon standing. **Step 2: synthesis of tert-butyl (2-methyl-3-oxo-3-(phenethylamino)propyl)carbamate**. A solution of 3-((tert-butoxycarbonyl)amino)-2-methylpropanoic acid (5 g, 24.6 mmol) and phenethylamine (3.4 mL, 27.06 mmol) in THF (200 mL) was cooled to 0 °C and treated with pyridine (2.19 mL, 27 mmol) and 1-(3-dimethylaminopropyl)-3-ethylcarbodiimide hydrochloride (EDCI, 5.66 g, 29.52 mmol). After the addition was completed, the ice bath was removed and the solution was stirred an additional 16 h at room temperature. Water (200 mL) was added and the solution was extracted with ethyl acetate (3x). The combined organic layers were dried over magnesium sulfate, filtered and concentrated. Purification via flash column chromatography (ethyl acetate in hexanes) gave tert-butyl (2-methyl-3-oxo-3-(phenethylamino)propyl)carbamate (3.31 g, 44% yield) as a white solid. **Step 3: synthesis of 3-amino-2-methyl-N-phenethylpropanamide**. While under a nitrogen atmosphere, an ice-cooled solution of *tert*-butyl N-[2-methyl-2-[(2-phenylethyl)carbamoyl]ethyl]carbamate (3.31 g, 10.8 mmol) in anhydrous dichloromethane (75 mL) was carefully treated with trifluoroacetic acid (25 mL, 336.6 mmol) and stirred for 2 h. The solution was concentrated, dissolved in chloroform (30 mL) and washed with saturated aqueous K_2_CO_3_. The resulting solution was re-concentrated to give 3-amino-2-methyl-N-phenethylpropanamide (2.23 g, 100%). **Step 4: synthesis of (2 R)-2,4-dihydroxy-3,3-dimethyl-N-(2-methyl-3-oxo-3-(phenethylamino)propyl)butanamide**. Under a nitrogen atmosphere, a solution of 3-amino-2-methyl-N-(2-phenylethyl)propanamide (1.9 g, 9.2 mmol) and pantolactone (1.09 g, 8.4 mmol) in methanol (20 mL) was stirred at 40 °C for 16 hr. The solution was then concentrated and subsequently purified using flash column chromatography (2–10% methanol in dichloromethane) to give (2 R)-2,4-dihydroxy-3,3-dimethyl-N-[2-methyl-2-[(2-phenylethyl)carbamoyl]ethyl]butanamide (1.92 g, 68% yield) as a waxy solid.

### NMR and mass spectrometry validation

^1^H NMR (400 MHz, DMSO-*d*_6_) δ 7.92 (t, *J* = 5.6 Hz, 1 H), 7.56 (dt, *J* = 42.2, 6.0 Hz, 1 H), 7.38–7.03 (m, 5 H), 5.36 (dd, *J* = 14.9, 5.6 Hz, 1 H), 4.45 (td, *J* = 5.6, 2.2 Hz, 1 H), 3.72 (dd, *J* = 5.6, 2.6 Hz, 1 H), 3.45–2.94 (m, 4 H), 2.70 (ddt, *J* = 10.4, 5.8, 1.8 Hz, 2 H), 2.51 (p, *J* = 1.9 Hz, 1 H), 0.96 (d, *J* = 6.9 Hz, 3 H), 0.90–0.63 (m, 6 H); HPLC using an Agilent 1100 equipped with an Eclipse + C18 4.1 × 55 mm 1.8-micron column and a flow rate of 0.5 mL/min eluting with a 5 to 100% gradient of acetonitrile in water modified with 0.1% formic acid over 9 minutes provided a t_R_ = 5.93 min and 100% purity as detected at 220 and 254 nM. Mass was determined using an Agilent 6120 quadrupole mass spectrometer M + 1^+^ = 337.2.

### *P. falciparum* strains and growth assays

*Plasmodium falciparum* (3D7, HB3, NF54 and W2) strains used in this study were obtained from MR4. Parasites were cultured as previously described^5^ in complete RPMI supplemented with Albumax and synchronized using three successive 5% sorbitol treatments^32^. A stock solution of α-PanAm was prepared at 1 mM in 10% dimethyl sulfoxide (DMSO) and was added to each well at increasing concentrations from 10 nM to 1 µM. A stock solution of pantothenic acid was prepared at 1 mM in sterile water and was added to each well for the complementation assay at the indicated concentrations. The growth assays to examine the susceptibility of parasites to α-PanAm were performed in 96-well plates with a starting parasitemia of 1% and total volume of 100 µl. Growth was assessed using the SYBR Green assay method as described by Johnson *et al*.^33^. Values are means ± standard deviation of growth assays performed in triplicates.

### *P. chabaudi chabaudi* AJ strain blood stage growth assay

Mice used in this study were female Swiss Webster (SW) (5 weeks old) purchased from Envigo Research Models and Services (Indianapolis, Indiana). Animal handling was conducted according to an approved protocol by the Institutional Animal Care and Use Committee (IACUC) of Tulane University. *P chabaudi chabaudi* AJ lethal strain was used to infect groups of three SW mice by intravenous inoculation. Growth of blood stage parasites was evaluated as previously described^27,34,35^. Doses of α-PanAm were prepared in 10% DMSO/PBS and injected IP daily for three consecutive days at a concentration of 40 mg/kg or 100 mg/kg per mouse.

### *In vitro* liver stage Growth Assay

Mosquito infections with *P. berghei* ANKA were conducted as previously described^36^. Hepatoma HepG2-CD81 cells were seeded in eight-well chamber slides at a density of 40,000 cells per well 48 hours prior to the assay. A total of 25,000 salivary gland sporozoites (day 18 after mosquito feeding) of *P. berghei* ANKA strain were re-suspended in DMEM-F12 medium (Gibco) supplemented with 3% Bovine Serum Albumin (Sigma) and added to each well. The sporozoites were incubated with the cells at 37 °C for 1 hr after centrifugation at 300 × g for 3 min. The cells were washed twice with PBS to remove all non-invading sporozoites and mosquito debris. 2.5 µM α-PanAm was added to DMEM-F12 medium supplemented with 10% Fetal Calf Serum (Gibco) and 1% Penicillin/Streptomycin (Gibco) and added to wells 2 hrs after sporozoite invasion. At 44 hours after invasion, all wells were fixed in methanol at room temperature for 5 min and then blocked in 10% FCS/PBS at 4 °C overnight. Primary antibodies against *P. berghei* HSP70 were diluted to 1:200 in 10% FCS/PBS and incubated with the cells for at 37 °C. Alexa Fluor 488-conjugated secondary anti-mouse was used to detect the bound primary antibodies. Nuclear staining performed with Hoechst dye (diluted 1:5000) was added to the mounting solution. Preparations were analyzed at a magnification of 400X by fluorescence microscopy.

### Yeast strains and growth assays

Yeast strains used in this study include: wild type MATa (BY4741: MATa his3Δ1 leu2Δ0 met15Δ0 ura3Δ0), MATα (BY4742: MATα his3Δ1 leu2Δ0 lys2Δ0 ura3Δ0), cab1^ts^ (JS31.13-24: *MATa ura3 his3 cab1ts*), and pan6Δ (MATα ura3Δ0, leu2Δ0 lys2Δ0 his3Δ1 Δpan6::HIS3). Wild type and mutant strains were propagated either in rich YPD medium composed of 2% bacto-peptone (Difco), 2% D-(+)-glucose (Sigma), 1% yeast extract (Difco), and if desired 2% agar (Sigma); or defined pantothenic acid –free medium composed of Yeast Nitrogen Base (YNB) lacking pantothenic acid and Complete Supplement Mixture (CSM) from MP Biomedicals. Where indicated media were supplemented with pantothenic acid prepared at 10 mM in sterile water to provide indicated concentrations. A stock solution of 20 mM α-PanAm was prepared in 10% dimethyl sulfoxide (DMSO), and added to each culture medium at the indicated concentrations. Growth assays were performed in 96- well plates in triplicates, with 150 μl medium per well inoculated with 10^4^ cells/ml. α-PanAm and fluconazole were diluted in 10% DMSO and were added to the plates to form a gradient of two-fold dilutions. α-PanAm ranged from 10 μg/ml to 640 μg/ml in WT and Pan6Δ, and from 0.156 μg/ml to 20 μg/ml in Cab1^ts^. Fluconazole ranged from 2 μg/ml to 32 μg/ml in all strains. The plates were then incubated at 30 °C. At the indicated time points, the 96 well plates were measured for absorbance (A_630_) using a BioTek SynergyMx microplate reader. Data were plotted in GraphPad Prism 7 as A_630_ over time for each concentration of pantothenic acid in each strain. The data for the timepoint where the untreated control for each pantothenic acid concentration reached growth saturation, or an optical density of around 1, was then used to calculate percent inhibition. The mean of the untreated control (0% inhibition) was calculated and used in the formula % inhibition = 100 − ((x/mean of untreated) * 100), where x = the data point of interest. The resulting data were plotted in GraphPad Prism and the non-linear regression function was used to generate a best-fit curve for percent inhibition, which also provides a best-fit value for MIC_50_. Data are expressed as mean ± SD of triplicates. Limiting dilution assays were performed on solid media by spotting 5 μl cell suspensions serially diluted ten-fold (10^6^–10^3^).

### Pantothenate kinase enzyme assay (radioactive assay)

Cell-free extracts from yeast were obtained by homogenization using glass bead beating, followed by centrifugation at 1000 rpm for 5 min. The pantothenate kinase assay was performed as previously described^37,38^. Briefly, the enzyme reaction contained the substrate D-[1-^14^C] pantothenate (2 nmol, 0.1 µCi), 72 μg protein as cell free extracts, and indicated concentrations of α-PanAm, in the reaction buffer (100 mM Tris/HCl, 2.5 mM MgCl_2_, 2.5 mM ATP, pH 7.4) in 40 μl. After incubation at 30 °C for 10 min, the reaction was arrested by adding 4 µl of 10% acetic acid. The reaction mixture was transferred to a spin column that contained two DE-81 ion-exchange filter disks (0.6mm in diameter). After 5 min, the spin column was centrifuged for 20 seconds at 1000 rpm to remove unreacted pantothenic acid. The spin column was washed three times with 1% acetic acid in ethanol. The radioactivity of the 4′-phosphopantothenate bound to the dried filter was quantified by liquid scintillation spectrometry. Pantothenate kinase activities are reported as enzyme specific activity (pmol of 4′-phosphopantothenate/mg-protein/min); and relative activities in the presence of α-PanAm.

### Analysis of Epitope-tagged CAB1 and cab1G351S fusion proteins expressed in *E. coli*

*Rosetta DE3* strains harboring pET28a(+) empty vector, pET28a(+)-His_6_-CAB1, pET28a(+)-His_6_-cab1^G351S^ were grown to saturation overnight in 3 ml LB-0.2% glucose, kanamycin (50 µg/ml) and chloramphenicol (34 µg/ml). The expression of the enzymes resulted in N-terminal His6 chimeras. The resultant cultures were diluted 100-fold in fresh medium, and grown to A_600_ ~0.5 at 30 °C. Expression of His_6_-CAB1 and His_6_-cab1G51S was induced by addition of 0.3 mM isopropylthiogalactoside (IPTG) followed by growing strains for 2 hrs at 37 °C, 2.5 hrs at 30 °C, or overnight at 22 °C. The cells were harvested by centrifugation (10,000 × g × 2 min, 4 °C), after the indicated times of induction and washed by resuspension in water and re-centrifugation. The cells were re-suspended in 500 μl 20 mM Tris-HCl, pH 7.4, 200 mM NaCl, 1 mM EDTA, and 10 mM beta-mercaptoethanol (β-ME), frozen in a dry ice-ethanol bath, stored overnight at −20 °C and subsequently thawed on ice. Cells were disrupted by sonication in an ice water bath, with a Fisher Sonic Dismembrator 500 (15 second burst at 20% amplitude, 3 times, with 30 second cooling intervals). Supernatants (20,000 × g × 20 min) and pellets were obtained by centrifugation. Distribution of His_6_-CAB1 and His_6_-cab1G351S between the supernatant and pellet fractions was monitored by western blot analysis of volume normalized samples, using anti- His_6_ antibody diluted 5,000 fold. For inhibitor studies, purified His_6_-CAB1 and His_6_-cab1^G351S^, were prepared from 200 ml of cultures after IPTG induction overnight at 22 °C. Cells were harvested by centrifugation at 5,000 × g × 20 min, and washed by re-centrifugation. The cells were resuspended in 5 mls 20 mM Tris-HCl, pH 7.4, 200 mM NaCl, 1 mM EDTA, and 10 mM β-ME, and sonicated in an ice- water bath (15 second bursts at 20% amplitude, 8 times, with 30 second cooling intervals). A 20,000 × g supernatant was prepared by centrifugation of the cell sonicate. Enzyme from supernatants were purified by Ni^2+^-nitrilotriacetic acid (Ni-NTA) affinity chromatography (Qiagen) following the manufacturer’s protocol with modifications. Briefly, 20,000 × g-supernatant fractions were incubated with Ni-NTA agarose in column buffer (6.4 mM imidazole, 50 mM potassium phosphate, 0.3 M NaCl, 10% glycerol, pH 7.4) with gentle agitation for 1 hr at 4 °C. Following enzyme adsorption, the affinity matrix was transferred to a column for elution. The unbound proteins were removed by washing the column with the buffer containing 6.4, 12.8, 32, or 64 mM imidazole, successively. Finally, the His_6_-CAB1 and His_6_-cab1G351S were eluted with buffer containing 128 mM imidazole. Imidazole was removed by gel filtration with a PD10 column (GE healthcare) using homogenization buffer. Enzyme purity was ~70% with respect to total protein. For activity measurements with the purified enzymes, 0.5 µg of His_6_-CAB1 and 2.5 µg of His_6_-cab1^G351S^ were incubated with 2 nmol D-[1-^14^C] pantothenic acid (0.01 µCi) in the pantothenate kinase reaction buffer in a volume of 40 μl.

### Kinase-Glo Plus Luminescent Kinase and ADP-Glo Kinase assays

The amount of ATP remaining in solution following kinase reaction was measured using the Kinase-Glo Plus luminescent kinase (Kinase-Glo) assay. Kinase assays were carried out at room temperature in 96-well plates. α-Pan Am at a concentration of 10 µM was used in the assay. For Kinase-Glo assay, buffer consisted of 100 mM Tris-HCl pH 7.4, 10 mM MgCl_2_, 100 μM pantothenate (PA), 100 μM ATP and 0.5 mg/ml γ- globulin. Kinase reactions were started with the addition of 500 ng active Cab1 or heat inactivated Cab1 (Cab1 subjected to 80 °C for 20 min) followed by incubation at room temperature for 1 hr. At the end of incubation, an equal volume (10 µl) of Kinase-Glo reagent was added to each sample. The plates were further incubated for 5 min prior to recording the luminescence. Reactions in the absence of ATP (background luminescence) denoted as negative controls while those in the presence of heat inactivated CAB1 (highest luminescence) denoted as positive controls. To calculate activation or inhibition of CAB1 by a compound, the following formula was used: % Inhibition = ((sample − negative control)/(positive control − negative control)) × 100. ADP-Glo Kinase assay was carried out in two steps after the completion of kinase reaction; removal of remaining ATP and conversion of enzyme reaction product ADP to ATP combined with luciferase/luciferin reaction. Briefly, the assay was performed in kinase buffer containing 40 mM Tris (pH 7.4), 20 mM MgCl_2_, 0.1 mg/ml BSA, 100 µM PA and 100 μM ATP. The reaction was started with the addition of active 500 ng Cab1 or heat inactivated Cab1 and incubated at room temperature for 1 hr. 5 μl of ADP-Glo kinase reagent was added to arrest the reaction and deplete the unconsumed ATP, leaving only ADP and a very low background of ATP. The samples were further incubated at room temperature for 40 min after which 10 μl kinase detection reagent was added to convert ADP to ATP and introduce luciferase and luciferin to detect ATP. LCMS of enzyme reactions were performed using an Agilent 1260 equipped with an Eclipse + C18 4.6 × 50 mm 1.8-micron column and a flow rate of 0.5 mL/min eluting with a 5 to 100% gradient of acetonitrile in water modified with 0.1% formic acid over 9 minutes. Mass was determined using an Agilent 6120 quadrupole mass spectrometer in both positive and negative ion mode. Specific masses were analyzed for PA-1 (mass range 217–219), P-PA-1 (mass range 297–299), in the negative ion mode while a-PanAm + 1 (mass range 336–338) was found reliably in the positive ion mode.

### Statistical analysis

All data analysis, graphing, and statistics were performed in Prism 7.0 software (GraphPad Software Inc., CA) or in Microsoft Excel 2016.
